# Epidemiology of herpes zoster at a tertiary care centre in Eastern India

**DOI:** 10.6026/973206300221085

**Published:** 2026-02-28

**Authors:** Sudipta Biswas, Koushik Biswas, Doyir Bagra, Kaushik Shome, Tanusree Sarkar

**Affiliations:** 1 Department of Dermatology, Venereology and Leprosy, Burdwan Medical College and Hospital, Burdwan, India; 2 Department of Biochemistry, All India Institute of Medical Sciences, Raebareli, India; 3 Department of Dermatology, Venereology and Leprosy, Prafulla Chandra Sen Government Medical College and Hospital, Arambagh, India

**Keywords:** Herpes zoster (HZ), post-herpetic neuralgia, varicella zoster virus (VZV), clinico-epidemiological study, India

## Abstract

Herpes zoster infection is characterized by dermatomal distribution of painful vesicles occurring due to reactivation of the latent varicella-zoster virus and poses the risk of serious complications. Therefore, it is of interest to investigate the epidemiology, clinical presentation and outcomes of patients diagnosed with herpes zoster in a tertiary care centre in Eastern India. From June 2023 to December 2024, a series of herpes zoster patients consulting the Dermatology Outpatient Department at Burdwan Medical College and Hospital participated in this cross-sectional study. Detailed history, clinical examination, Tzanck smear, hemogram, fasting plasma glucose and HIV screening were conducted. The significance of early diagnosis, prompt antiviral therapy and appropriate pain management in reducing complications such as postherpetic neuralgia and secondary infections is shown in this study.

## Background:

Herpes zoster (HZ) or shingles, typically presents as a localized, painful and vesicular rash confined to a single dermatome, which is the area of skin supplied by a single spinal or cranial nerve [1]. This characteristic rash is usually preceded by pain, tingling or burning sensations in the affected region, making it distinct from other dermatological conditions. The rash often persists for two to four weeks, with symptoms ranging from mild discomfort to severe, debilitating pain [2]. This disease occurs due to reactivation of the varicella-zoster virus (VZV) [3]. After an initial chickenpox infection, the VZV remains dormant within the sensory ganglia of the cranial nerve or the dorsal root ganglia of the spinal nerve, which can reactivate years later, under immune-compromised conditions [4]. With ageing, natural immunity declines due to a decline in cell-mediated immunity. Further immune-suppressive conditions viz. cancer chemotherapy or use of immunosuppressive drugs; increase the risk of HZ [5]. Stress and physical trauma may also play contributory roles. As the immune system weakens, the latent virus travels along the nerve fibres to the skin, causing inflammation and characteristic HZ rash. As immunity decline with age, HZ predominantly affects older adults [6]. Complications arising from HZ are serious and may extend beyond the initial painful rash. Post-herpetic neuralgia (PHN) is a common sequelae in elderly patients, characterized by persistent lancinating or burning pain in the affected dermatome that persists for three months or over, after the onset of infection [7]. It often leads to significant physical limitations, as patients may experience difficulty performing everyday tasks, contributing to increased dependence on caregivers and affecting overall well-being [8]. PHN often leads to emotional and psychological distress, frustration, anxiety and depression, as patients struggle with feelings of helplessness due to the chronic nature of their condition. Sleep disorders are common, as the pain often disrupts normal sleep patterns [9]. Poor-quality sleep leads to daytime fatigue. PHN comprehensive pain management requires both physical and psychological support [10]. Severe HZ cases may lead to motor nerve involvement, resulting in limb paralysis or affect the cranial nerves, potentially causing visual or auditory complications [11]. Cardiovascular complications like myocarditis, an inflammation of the heart muscle and cerebrovascular events have also been linked to virus-induced inflammatory responses. Scarring and pigmentary alterations from HZ can have both physical and psychological impacts [12]. Herpes zoster ophthalmicus (HZO) occurs when the VZV reactivates along the ophthalmic branch of the trigeminal nerve. Its symptoms include redness, swelling, pain in the eye, sensitivity to light and blurred vision. Untreated cases may result in serious complications such as keratitis, uveitis and even permanent vision loss due to corneal scarring or optic nerve damage, highlighting the importance of prompt diagnosis and treatment of HZO [13]. Commonly prescribed antiviral medications like acyclovir, famciclovir or valaciclovir inhibit VSV replication, shortening the duration of symptoms and lowering the risk of complications. Initiating antiviral therapy within 72 hours of rash onset is particularly beneficial, as it can significantly reduce the intensity and duration of the acute phase [14]. Topical applications, such as calamine lotion or cold compresses, can provide soothing relief and help reduce itching and inflammation of the rash. Hygiene and protective dressings are essential for preventing bacterial super infection of the lesions, which may lead to scarring [15]. Pain management in PHN typically begins with basic analgesics, such as acetaminophen or nonsteroidal anti-inflammatory drugs (NSAIDs), but severe cases may require stronger opioid-based pain relief [14]. Opioids are generally used cautiously due to potential side effects, including dependency and gastrointestinal symptoms. For severe cases, medications such as anticonvulsants (e.g., gabapentin, pregabalin) and tricyclic antidepressants, which help modulate nerve pain pathways, are prescribed. Topical therapies, including lidocaine patches and capsaicin creams, are also effective for localized pain relief [16, 17]. Therefore, it is of interest to investigate the epidemiology, clinical presentation and outcomes of patients diagnosed with herpes zoster in a tertiary care centre in Eastern India.

## Methods:

## Study design:

This descriptive cross-sectional study was conducted at the Department of Dermatology, Venereology and Leprosy, Burdwan Medical College and Hospital, Burdwan, a tertiary care centre in Eastern India. The study was conducted over 18 months (June 2023-December 2024). Ethical clearance was taken from the Institutional Review Committee before starting the study (Memo no. BMC/IEC/240).

## Study participants:

Inclusion criteria were all clinically diagnosed HZ patients who were willing to participate in the study. Patients with complicated HZ, like visceral involvement, were excluded from the study. Patients who met the inclusion and exclusion criteria were identified during consultation in the Dermatology OPD and they were informed about the research by the study team.

## Sample size:

Based on a previous study by Yang *et al.* [18], which reported a HZ prevalence of 7.7% and applying the formula, n= z^2^ p (1- p)/ d^2^, where n is the necessary sample size, z the level of confidence at 95% (1.96 standard value), p the prevalence and d the error margin at 5% (0.05 standard value), we calculated the sample size to be 109. Considering a 10 % loss to follow up, we finally took 125 patients for this study.

## Investigations:

HZ diagnosis was based on clinical examination and if necessary Tzanck smear (Giemsa stain) examination. Tzanck smear was useful in cases where the clinical presentation was inconclusive. Detection of ballooned epithelial cells and multinucleated giant cells in Tzanck smear were indicative of herpes virus infection. Although not specific to HZ alone, these findings were suggestive of viral reactivation within the affected dermatome and supported the clinical diagnosis when combined with other symptomatic and laboratory findings. Other routine tests which were done for patient evaluation included a complete hemogram, fasting plasma glucose and HIV antibodies test. Complete hemogram was done in a five-part cell counter. Estimation of fasting plasma glucose was carried out by the glucose oxidase peroxidase method using a fully automated chemistry analyzer. HIV antibodies screening was done by ELISA (enzyme-linked immunosorbent assay) kits.

## Study procedure:

Before enrollment, the research team explained the study details to the patients, providing a patient information sheet that outlined the study's objectives, methods and potential benefits and risks. Following informed consent, a pre-designed case report form was used to record the data of each patient. The data included information on chief complaints, history of present illness, past medical history, family history, birth history and drug history. Thereafter, patients underwent general and systemic examination. A dermatological examination was performed to evaluate skin lesions and map the dermatomal involvement. Samples were sent to the laboratory for Tzanck smear test, complete hemogram, fasting plasma glucose and screening of HIV antibodies. All patients were followed up weekly during the first month and monthly thereafter, till four months. The data collected at each follow-up session were recorded in the patient's case report form.

## Statistical analysis:

We maintained strict confidentiality throughout the study regarding the patient data. All patient data were recorded and analyzed by using a Microsoft Excel spreadsheet. We presented all the categorical data as frequencies and percentages.

## Results:

The demographic characteristics of 125 HZ patients enrolled in this study are presented in Table 1. The median age of patients was 46 years (range 6 - 78 years). The majority of patients (n=55, 44 %) belonged to the 40-60 years age group. Fifty-five (44%) patients were female, while 70 (56%) were male. Based on religious practice, 82 (65.6%) were Hindu and 43 (34.4%) were Muslim.

Table 2 presents the distribution of patients based on the day of their first visit to the Dermatology OPD from the onset of HZ symptoms. Six (4.8 %) patients sought medical attention on the second day of illness, indicating that early consultation was rare. Out of 125 patients, 49 (39.2 %) presented to the OPD on the fourth day from the onset of symptoms, followed by 32 (25.6 %) on the fifth day. This may be because the majority of patients sought medical consultation after experiencing persistent or worsening symptoms, probably due to increasing pain or lesion progression. All patients in our study had dermatomal involvement. The distribution of dermatomal involvement observed was 19 (15.2 %) cervical, 69 (55.2 %) thoracic, 17 (13.6 %) lumbar, 2 (1.6 %) sacral and 18 (14.4 %) trigeminal. Thus, the majority of the patients in our study had involvement of the thoracic dermatome. Among the 125 patients, 81 (64.8 %) experienced dermatomal pain, 28 (22.4 %) had itching, 23 (18.4 %) had fever and 16 (12.8 %) had paraesthesia. The clinical features of patients are presented in Table 3. Out of 125 patients, 87 (69.6 %) had a documented history of chickenpox, while 38 (30.4 %) did not recall having a past infection. Complications from HZ developed in 37 (33.03 %) patients. Out of them, 20 (16 %) had PHN, 8 (6.4 %) had scarring, 5 (4 %) had secondary infection and 4 (3.2 %) had keloid. Table 4 presents the occurrence of complications from HZ. Lastly, we analysed the risk factors of HZ in our patients. Among the 125 patients, nine (7.2 %) had diabetes mellitus, four (3.2 %) were HIV-positive, two (1.6 %) had malignancy, one (0.8 %) had pulmonary tuberculosis and one (0.8 %) was under steroid therapy. No risk factor was noted in 108 (86.4 %) patients. Table 5 presents the prevalence of risk factors among patients in our study.

## Discussion:

In our study, 55 (44 %) of patients were in the 41-60 years age group, followed by 41 (32.8 %) in the 21-40 years age group and the median age of patients was 46 years. The mean age of HZ patients in a study by Goh and Khoo [19] was 48.8 years. Immunosenescence associated with ageing leads to a decline in cell-mediated immunity, thereby facilitating the reactivation of latent VZV in older individuals [20]. Numerous Indian studies have reported that the disease is becoming more common among the young [21,22-23]. Mass vaccination initiatives and the immunity status of individuals during the primary infection are reported to be linked to this trend [24]. Our finding of a male predominance in HZ cases in this study (56% males vs. 44% females) is consistent with previous studies [21, 23 and 25]. This higher prevalence in males has been attributed to lifestyle-related risk factors like smoking, alcohol consumption and occupational stress, which can contribute to immune modulation and increase susceptibility to viral reactivation [26]. The delay in seeking medical consultation for HZ, as observed in this study, is a significant concern, as early antiviral treatment is most effective when initiated within 72 hours of symptom onset. This delay may be due to multiple factors, viz., lack of awareness about the disease, misinterpretation of initial symptoms as minor skin irritation or reliance on home remedies before seeking professional medical advice [27]. Our finding of a predominance of thoracic dermatome involvement aligns with previous research findings [21, 23, 25 and 28]. This may be attributed to the larger surface area of these dermatomes and their extensive innervation, which increases the likelihood of viral replication and symptomatic manifestation. Trigeminal involvement can lead to HZO, which poses a risk of vision impairment if not promptly treated [29]. The rare occurrence of sacral involvement in two (1.6%) patients suggests that these regions may be less prone to viral reactivation, possibly due to differences in ganglionic viral load or immune surveillance [30]. However, when these areas are affected, the presentation may be atypical, potentially delaying diagnosis and increasing the risk of complications such as neurogenic bladder dysfunction [31].

Eighty-one (64.8 %) patients in our study had dermatomal pain. Pain preceding or accompanying the rash is often the first clinical indication of HZ and its intensity varies widely among patients. Several factors, including age, immune response and pre-existing neurological conditions, contribute to this variability [5]. Early identification and treatment of dermatomal pain reduces the severity and duration of zoster-associated pain, as well as the risk of PHN [16]. The absence of dermatomal pain in 44 (35.2%) patients suggests that HZ can present atypically, necessitating careful clinical evaluation beyond pain assessment alone. Some individuals may have mild or subclinical viral reactivation, leading to less pronounced nerve irritation. Additionally, differences in pain perception, especially in elderly patients, may result in underreporting of symptoms [31]. In this study, only 20 (16 %) patients were affected by PHN. This may be due to early intervention and appropriate pain management therapy. Only five (4 %) patients in this study had secondary bacterial infections. Lee *et al.*. [32] reported a 5 % incidence of secondary infection in HZ patients in their study. This highlights that despite the presence of open vesicular lesions, the majority of HZ cases can heal without significant complications if patients receive early intervention and appropriate care. Moreover, antiviral therapy reduces the viral load, potentially limiting the duration of the vesicular phase and decreasing the risk of bacterial colonization. Nine (7.2 %) patients in our study had diabetes mellitus. Papagianni *et al.*. [33] reported that diabetes is linked to a higher rate of PHN and other complications, making early diagnosis and management crucial in this population. Four (3.2 %) patients in our study screened positive for HIV antibodies. Risk factors such as diabetes, HIV, malignancy and immunosuppressive therapy emphasize the importance of early screening and preventive measures. Clinicians should consider the use of the zoster vaccine, which has been shown to reduce the incidence and severity of HZ in immunocompromised individuals, including those with diabetes, HIV, malignancy or on immunosuppressive therapy [34]. Affected patients should be counselled to refrain from contacting those who did not have varicella in the past or who have not received the varicella vaccine [35]. Serology screening and vaccination of pregnant women, besides including the vaccine in National Immunization Programme of India will be helpful in future [36]. At present, HZ is not a notifiable disease in India. Declaring it a notifiable disease can increase surveillance, raise awareness and reduce morbidity [37]. Our study was carried out in a tertiary-level hospital in Eastern India. Hence, the findings may not be generalizable to other settings, particularly rural or primary healthcare facilities, where access to timely medical intervention may differ. The Tzanck smear used in this study may have false negatives due to sampling errors, low viral load or examination of lesions in later stages of healing when fewer infected cells are present. Polymerase chain reaction or direct immunofluorescence assays for definitive confirmation and serological confirmation of past VZV exposure were not available in our setting. Multi-centric study with serological test provision of past VZV exposure may provide a more clinico-epidemiological pattern of the disease in this region.

## Conclusion:

The significance of early diagnosis, prompt antiviral therapy and appropriate pain management in reducing complications such as postherpetic neuralgia and secondary infections is shown. The majority of patients had no identifiable risk factors, reinforcing the fact that HZ can occur even in immunocompetent individuals, although those with diabetes, HIV or immunosuppressive conditions are at a higher risk. Given the burden of HZ and its potential complications, preventive strategies such as vaccination play a crucial role in reducing disease incidence and severity, particularly in high-risk populations.

## Financial support and sponsorship:

Nil|

## Figures and Tables

**Table 1:** Demographic characteristics of study patients

**Characteristics**		**n (%)**
	< 20 years	4 (3.2)
Age group	20 - 40 years	41 (32.8)
	40 - 60 years	55 (44)
	≥ 60 years	25 (20)
	Male	70 (56)
Sex	Female	55 (44)
Religion	Hindu	82 (65.6)
	Muslim	43 (34.4)

**Table 2:** Distribution of study subjects as first visit to OPD from onset of symptoms

**Day of first visit to OPD from disease onset**	**n (%)**
2nd day	6 (4.8)
3rd day	18 (14.4)
4th day	49 (39.2)
5th day	32 (25.6)
6th day	10 (8)
7th day	8 (6.4)
8th day	2 (1.6)
Total	125 (100)

**Table 3:** Clinical features of the patients at presentation

**Clinical feature**		**n (%)**
Dermatome involvement	Cervical	19 (15.2)
	Thoracic	69 (55.2)
	Lumbar	17 (13.6)
	Sacral	2 (1.6)
	Trigeminal	18 (14.4)
	Total	125 (100)
Dermatomal pain		81 (64.8)
Itching		28 (22.4)
Fever		23 (18.4)
Paraesthesia		16 (12.8)

**Table 4:** Complications from herpes zoster in study patients

**Complication**	**n (%)**
Post herpetic neuralgia	20 (16)
Scarring	8 (6.4)
Secondary infection	5 (4)
Keloid	4 (3.2)
Total	37 (33.03)

**Table 5:** Distribution of risk factors in study patients

**Risk factor**	**n (%)**
Diabetes mellitus	9 (7.2)
HIV	4 (3.2)
Malignancy	2 (1.6)
Pulmonary tuberculosis	1 (0.8)
Steroid therapy	1 (0.8)
No risk	108 (86.4)
